# Environmental Conditions May Shape the Patterns of Genomic Variations in *Leishmania panamensis*

**DOI:** 10.3390/genes10110838

**Published:** 2019-10-24

**Authors:** Carlos M. Restrepo, Alejandro Llanes, Eymi M. Cedeño, Jim H. Chang, Jennifer Álvarez, Margarita Ríos, Homero Penagos, José A. Suárez, Ricardo Lleonart

**Affiliations:** 1 Centro de Biología Celular y Molecular de Enfermedades, Instituto de Investigaciones Científicas y Servicios de Alta Tecnología (INDICASAT AIP), Panama 0801, Panama; 2 Department of Biotechnology, Acharya Nagarjuna University, Guntur, Andhra Pradesh 522510, India; 3 Departamento de Biotecnología, Facultad de Ciencias de la Salud, Universidad Latina de Panamá, Panama 0801, Panama; 4 Escuela de Biología, Facultad de Ciencias Naturales, Exactas y Tecnología, Universidad de Panamá, Panama 0801, Panama; jenniferalvarez1412@gmail.com; 5 Unidad Clínica de Investigación, Clínica de Medicina Tropical, Instituto Conmemorativo Gorgas de Estudios de la Salud (ICGES), Panama 0801, Panama; 6 Hospital Regional Dr. Rafael Hernández, Caja de Seguro Social, Chiriquí 0401, Panama

**Keywords:** *Leishmania panamensis*, genomic variations, antimony resistance

## Abstract

Due to the absence of transcriptional regulation of gene expression in *Leishmania* parasites, it is now well accepted that several forms of genomic variations modulate the levels of critical proteins through changes in gene dosage. We previously observed many of these variations in our reference laboratory strain of *L. panamensis* (PSC-1 strain), including chromosomes with an increased somy and the presence of a putative linear minichromosome derived from chromosome 34. Here, we compared the previously described genomic variations with those occurring after exposure of this strain to increasing concentrations of trivalent antimony (Sb^III^), as well as those present in two geographically unrelated clinical isolates of *L. panamensis*. We observed changes in the somy of several chromosomes, amplifications of several chromosomal regions, and copy number variations in gene arrays after exposure to Sb^III^. Occurrence of amplifications potentially beneficial for the Sb-resistant phenotype appears to be associated with the loss of other forms of amplification, such as the linear minichromosome. In contrast, we found no evidence of changes in somy or amplification of relatively large chromosomal regions in the clinical isolates. In these isolates, the predominant amplifications appear to be those that generate genes arrays; however, in many cases, the amplified arrays have a notably higher number of copies than those from the untreated and Sb-treated laboratory samples.

## 1. Introduction

*Leishmania* is a kinetoplastid parasite causing several clinical presentations collectively known as leishmaniasis. The parasite develops as a flagellated promastigote in its sandfly vector and as an amastigote with a retracted flagellum in the macrophages of its vertebrate hosts. *Leishmania* species that are known to be pathogenic to humans are divided into two main subgenera, namely, *L.* (*Leishmania*) and *L.* (*Viannia*). Species belonging to the *L.* (*Viannia*) subgenus are exclusively present in Central and South America and cause mainly cutaneous leishmaniasis. However, in some cases, the parasites can migrate to the nasopharyngeal area and cause a more severe presentation called mucocutaneous leishmaniasis. The *L.* (*Viannia*) subgenus comprises two major species complexes, namely the *L. braziliensis* complex, which also include *L. peruviana*, and the *L. guyanensis* complex, which also include *L. panamensis*. *L. panamensis* is the main causative agent of leishmaniasis in Panama and it is responsible of a significant number of cases in neighboring countries.

As in other kinetoplastids, gene expression in *Leishmania* is not primarily regulated at the initiation of transcription, but mainly at the post-transcriptional level by mechanisms that modulate the amounts of mRNA and its stability [1]. Among these mechanisms, it has been demonstrated that cellular levels of critical proteins are modulated by changes in gene dosage [2]. These changes are in turn associated with different types of genomic variations typically involving some form of genetic amplification, such as the increase in copy number of individual genes to form tandem gene arrays, the amplification of larger regions in the form of linear or circular extrachromosomal episomes, as well as changes in the somy of entire chromosomes [3]. Both intra- and extrachromosomal amplifications are largely stochastic and appear to involve recombination of repetitive elements flanking the region to be amplified [4]. Variations in the somy of individual chromosomes are also stochastic in nature and can occur even in different individuals within the same population, giving rise to the phenomenon known as mosaic aneuploidy [5].

Despite their stochastic nature, many of these genomic variations appear to represent an adaptive advantage for *Leishmania* parasites. Perhaps the best characterized example are the variations selected in the presence of pentavalent antimonials [6]. These compounds are pentavalent antimony (Sb^V^) derivatives that have been used as the first-line treatment for leishmaniasis since the 1940s. After an obligate reduction step, Sb^V^ is converted to trivalent antimony (Sb^III^) [7], which can kill the parasite trough mechanisms that are not yet well understood. Genetic changes associated with resistance to Sb^III^ can appear quickly and transiently, often disappearing when the drug pressure is removed [8]. Different forms of genetic amplifications potentially associated with the acquisition of the Sb-resistant phenotype have been reported for species of the *L.* (*Leishmania*) subgenus, including *L. infantum* [9], *L. donovani* [10,11,12], and *L. major* [13], as well as for those belonging to the *L.* (*Viannia*) subgenus [14,15].

Variations in chromosome somy were ubiquitously observed in all these studies, but in some cases, the episomal amplification of relatively large chromosomal regions was also reported. Particularly relevant in this context is the amplification of a region of chromosome 23, called the H-locus [16], leading to circular episomes. This region contains at least one gene encoding an ATP-binding cassette (ABC) transporter known as multidrug resistance protein A (MRPA) or P glycoprotein A (PGPA), which has long been known to be overexpressed in resistant amastigotes [17,18]. In addition, deletion of chromosomal regions has also been reported, typically leading to gene loss or to a reduction in copy number. An example is the deletion of subtelomeric segments of chromosome 31 containing the AQP1 gene, which codes for a specific aquaglyceroporin that imports Sb^III^ into amastigotes [19].

Genomic amplifications have also been observed in *Leishmania* in absence of drug pressure. For instance, several types of amplicons deriving from a subtelomeric region of chromosome 34, called LD1, have been reported in different *Leishmania* species [20,21]. Since this region contains a gene encoding a biopterin transporter (BT1), its amplification may be associated with an improved acquisition of pterins required for folate metabolism. A particular type of LD1 amplification, consisting of a linear minichromosome of ~245 kb, was initially observed in some strains of *L. braziliensis* [22]. Later, we found evidenced of the presence of a similar minichromosome in our reference *L. panamensis* strain (strain PSC-1) [23], thus suggesting that this particular type of LD1 amplification may be specific to the *L.* (*Viannia*) subgenus. Here, we aimed to compare the patterns of genomic variations among the PSC-1 strain, an antimony-resistant mutant and two geographically unrelated clinical isolates of *L. panamensis*. In order to generate the resistant mutant, we exposed the PSC-1 strain to increasing concentrations of potassium antimonyl tartrate. Whole genome sequencing (WSG) was performed at seven different Sb^III^ concentrations, thus allowing us to characterize not only the genomic variations in the final resistant mutant but also the dynamics of these variations during acquisition of the resistant phenotype.

## 2. Materials and Methods

### 2.1. Ethical Statement

The work in human patients was approved by the ethical board of Hospital Punta Pacífica, Panama, with approval code FID14-126 No.27 and approval date of October 28, 2015. Although data were analyzed anonymously, written informed consent was obtained from patients before samples were taken.

### 2.2. Clinical Sample Collection

Sample collection was conducted at Instituto Conmemorativo Gorgas de Estudios de la Salud (ICGES), Panama City, Panama and Hospital Regional Dr. Rafael Hernández, Chiriquí, Panama. ICGES is the national reference center for clinical diagnosis of leishmaniasis and patients are referred to this institute from almost all over the country. Children less than six years of age, patients with lesions indicative of intercurrent bacterial or fungal infection and patients on active treatment for cutaneous leishmaniasis were excluded from the study. Cutaneous lesions were examined by a specialized physician for proper identification of the lesion and exclusion of secondary bacterial infection. Lesion aspirates were collected aseptically using a 25-gauge needle and disposable syringe containing 0.5 mL of sterile saline, which was inserted intradermally into the active border of the lesion. The syringe was rotated and tissue fluid was then aspirated into the needle. Aspirated fluid was inoculated into biphasic medium comprised of a solid phase of blood agar base (Sigma-Aldrich, St. Louis, MO, USA) supplemented with 50 µg/mL gentamicin (Sigma-Aldrich, St. Louis, MO, USA) and 10% of aseptically collected defibrinated rabbit blood, and a liquid phase of Schneider’s insect medium (Sigma-Aldrich, St. Louis, MO, USA) at pH 7.2, supplemented with 20% (*v*/*v*) heat-inactivated fetal bovine serum (Gibco, Gaithersburg, MD, USA) and 50 µg/mL gentamicin. Isolates were incubated at 25 °C and monitored microscopically for 15 days for the presence of promastigotes.

### 2.3. Parasite Culture and Generation of an Antimony-Resistant L. panamensis Strain

*L. panamensis* promastigotes were axenically maintained in serum-supplemented Schneider’s insect medium, prepared as described in the previous section, and incubated at 25 °C. The antimony-treated *L. panamensis* samples were selected from the wild type PSC-1 strain (MHOM/PA/1994/PSC-1) in 25 cm^2^ tissue culture flasks containing 10 mL of serum-supplemented Schneider’s medium in the presence of increasing Sb^III^ concentrations. Potassium antimonyl tartrate (≥ 99% purity) (Sigma-Aldrich, St. Louis, MO, USA) was used as the source of Sb^III^. Stepwise drug selection started from 50 µM up to 2000 µM of Sb^III^.

### 2.4. Antimony Susceptibility Assay

Resazurin sodium salt (7-hydroxy-3H-phenoxazin-3-one-10-oxide sodium salt) was employed for antimony susceptibility assay. Resazurin is a redox indicator that reduces to fluorescent resorufin as a result of the metabolic activity of cells. Mid-log growth phase promastigotes were diluted in serum-supplemented Schneider’s medium and seeded in a 96-well black plate (Thermo Scientific, Nunc, Rochester, NY, USA) at a density of 1 × 10^6^ parasites per well in a final volume of 100 µL. Parasites were incubated at 25 °C during 48 hours in presence or absence of several half-log dilutions of potassium antimonyl tartrate. After the incubation period, 20 µL of a 0.15 mg/mL solution of resazurin sodium salt in phosphate-buffered saline (PBS) (Sigma-Aldrich, St. Louis, MO, USA) were added to each well and the plate was incubated at 25 °C for 24 hours. Parasite viability was measured fluorometrically (λ_ex_ 560 nm; λ_em_ 590 nm) in a multidetection microplate reader (Synergy HT-Biotek, Winooski, VT, USA) and expressed in relative-fluorescent units (RFU). The assay was performed in triplicates and the half maximal effective concentration (EC_50_) was calculated by adjusting a sigmoidal dose–response curve using GraphPad Prism version 6.0 (GraphPad Software Inc., San Diego, CA, USA).

### 2.5. Whole Genome Sequencing

High-quality genomic DNA was extracted from the untreated PSC-1 strain and from the treated samples at each different Sb^III^ concentration (50, 100, 200, 400, 800, 1600, and 2000 µM Sb^III^), as well as from the two clinical isolates. Genomic DNA was extracted from stationary phase promastigotes, using a commercial salting out procedure as recommended by the manufacturer (Wizard Genomic DNA purification kit, Promega, Madison, WI, USA). Size, integrity, and presence of contaminants in the DNA samples were assessed through gel electrophoresis. DNA concentration was estimated by the picogreen method (Invitrogen, Carlsbad, CA, USA) using Victor 3 fluorometry (PerkinElmer, Waltham, MA, USA). DNA purity was measured using a NanoDrop 2000 spectrophotometer (Thermo Scientific, Waltham, MA, USA). Sequencing libraries with 150 bp shotgun (paired-end) reads were prepared from 100 ng of genomic DNA by using the TruSeq Nano sample preparation protocol (Illumina, Inc., San Diego, CA, USA) and then sequenced in a NovaSeq 6000 instrument for a total throughput of 4 Gb per sample.

### 2.6. Estimation of Chromosome Somy

Illumina reads were first normalized to a total of 14 million per sample and trimmed to 100 bp with Trimmomatic [24]. Reads were aligned to the reference genome by using BWA-MEM [25]. SAMtools depth (version 0.1.19) [26] was used to record the read depth per base for all chromosomes at the different Sb^III^ concentrations. Somy of individual chromosomes was estimated by first calculating the median read depth for each chromosome and then dividing this value by the mode of all calculated medians, which was expected to correspond to disomic chromosomes. Before computing the median read depth for each chromosome, all values lying ±3 standard deviations away from the mean were considered outliers and were further removed in order to avoid inaccurate estimations.

Since it has been suggested that large local variations in read depth [10,27] and chromosome length [3,10] can both affect somy estimates, we compared our original estimations with those obtained with the methodology proposed by Dumetz et al. [11]. This methodology is based on the same principle, but differs in that the median read depth is first computed by dividing each chromosome in 2500 bp bins and the somy estimation is done with a median calculated from its fourteen neighboring chromosomes, instead of using a single median value for the whole dataset. Statistical significance of the somy estimates were calculated by using the Mann–Whitney–Wilcoxon (MWW) test performed with the Statistics::Test::WilcoxonRankSum package from Perl (version 5.16). Samples with a *P* ≤ 10^−5^ were considered to be significant. The following ranges were used when assigning a somy to each chromosome from the corresponding estimates: disomic: ≥1.5, <2.5; trisomic: ≥2.5, <3.5; tetrasomic: ≥3.5, <4.5; pentasomic: ≥4.5, <5.5 and hexasomic: ≥5.5, <6.5.

### 2.7. Detection of Genetic Amplifications

Local amplifications within chromosomes were identified by using CNVnator [28] with default parameters. CNVnator results were complemented with manual revision to identify all local peaks spanning regions larger than 2 kb in the distribution of read depth along each chromosome.

### 2.8. Detection of Variants Associated with Protein-Coding Genes

Copy number variations (CNVs) associated with protein-coding genes were identified as described for entire chromosomes, but submitting an additional file to SAMtools with the corresponding annotations in BED format. The haploid copy number for each gene was then estimated by the ratio between its median read depth and the median read depth of its corresponding chromosome. In addition, the Genome Analysis Toolkit (GATK) (version 4.0.11) [29] was used to identify single nucleotide variants (SNVs) and small insertion/deletions (indels) of up to 3 bp, while SvABA [30] was used to identify larger indels and structural variants. For the GATK methodology, we separated the reads mapping to each chromosome into individual BAM files and submitted them independently to the UnifiedGenotyper module, so that the somy estimated for each chromosome could be incorporated as a parameter of the variant calling algorithm.

## 3. Results

### 3.1. Generation of an Antimony-Resistant L. panamensis Strain

**Figure 1:**
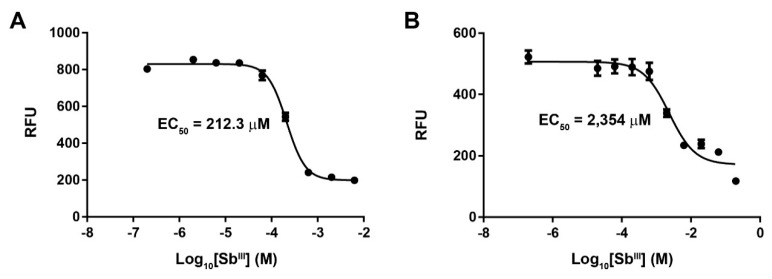
Variation of the EC_50_ value in a laboratory strain of *L. panamensis* exposed to increasing concentrations of antimony tartrate. Figure shows the dose–response curves for the untreated PSC-1 strain (**A**) and the final-stage parasite culture exposed to 2000 μM of Sb^III^ (**B**). Response is shown as relative fluorescence units (RFU) measured as part of the resazurin assay.

Antimony resistance was developed in vitro in our reference PSC-1 strain via exposure to increasing concentrations of potassium antimonyl tartrate. Susceptibility to Sb^III^ was measured through an indirect fluorescence assay with resazurin. The untreated strain showed an initial EC_50_ of 212.3 μM (Figure 1A). This strain was subjected to increasingly higher concentrations of Sb^III^ during a period of 30 weeks, starting at 50 µM and up to a maximum of 2000 μM. Resistant cultures were selected at 400, 800, 1600, and 2000 μM of Sb^III^, representing resistance indexes (RI) of 1.88, 3.76, 7.54, and 9.42, respectively. At each selection point, parasites were grown in the presence of the appropriate Sb^III^ concentration, during four passages, before DNA extraction and passage to the next Sb^III^ concentration. The final-stage culture showed an EC_50_ of 2354 μM (Figure 1B) and retained the resistant phenotype after 10 passages in absence of antimony.

In order to compare the findings observed in the laboratory strains, we also included in this study two *L. panamensis* clinical samples, isolated from human patients with no record of treatment failure. The Sb^III^ susceptibility assay for these isolates, named BD-02 and RG-01, revealed EC_50_ values of 26.58 μM and 5.27 μM, respectively. Surprisingly, both values are notably lower than the initial EC_50_ measured for our PSC-1 reference strain, suggesting that this laboratory-adapted strain may have a lower susceptibility than natural isolates, although it does not exhibit a defined Sb-resistant phenotype.

### 3.2. Variations in Chromosome Somy and Relatively Large Chromosomal Regions

**Figure 2:**
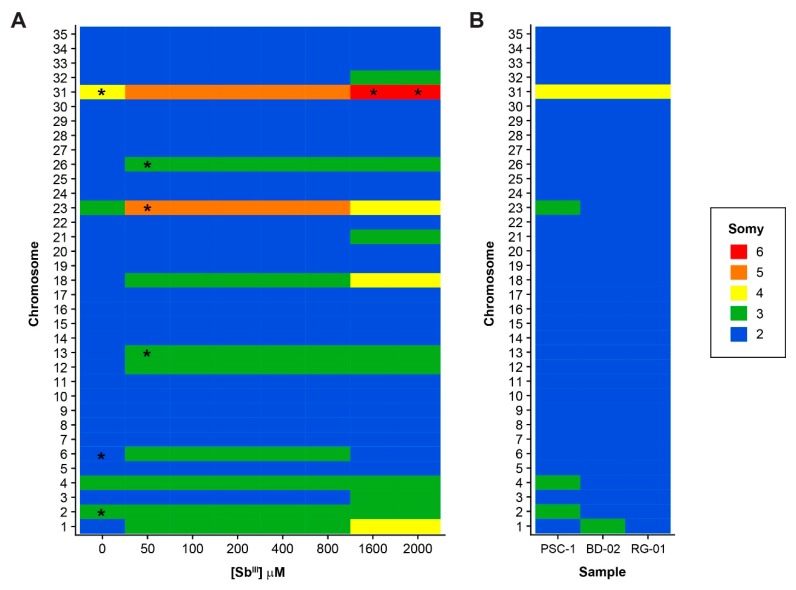
Variations in chromosome somy among all the samples included in this study. Estimates of the somy of each individual chromosome are shown for Sb-treated samples (**A**) and clinical isolates (**B**). The untreated PSC-1 strain is included as a control in both panels. Asterisks (*) indicate samples with values suggesting “intermediate” somy, defined as those estimated to be 0.5 units higher than the lower nearest integer by at least one of the methodologies used. In the heatmaps, values were approximated to the nearest integer to simplify the figure, using the following ranges: **2**: ≥1.5, <2.5; **3**: ≥2.5, <3.5; **4**: ≥3.5, <4.5; **5**: ≥4.5, <5.5 and **6**: ≥5.5, <6.5.

Whole genome sequencing (WGS) was performed for 11 *L. panamensis* samples, namely, the untreated PSC-1 strain, the antimony-treated samples collected at seven different Sb^III^ concentrations, and the clinical isolates BD-02 and RG-01. Sequence data generated in this study was submitted to the Sequence Read Archive (SRA) and is accessible through BioProject PRJNA558039. Variations in chromosome somy and in relatively large chromosomal regions were inferred from the distribution of sequence reads, after mapping them to our *L. panamensis* reference genome [23]. Chromosome somy was estimated by first using the mode of the median read depth for all chromosomes and then applying the strategy suggested by Dumetz et al. [11], with similar results in both cases (Table S1). Eleven chromosomes (1, 3, 6, 12, 13, 18, 21, 23, 26, 31, and 32) showed at least one statistically significant change in somy at a different Sb^III^ concentration during acquisition of the Sb-resistant phenotype, when compared to the untreated PSC-1 strain (Figure 2A; Table S2). Three of these, in fact, showed a marked increase in somy apparently correlating with the increase in Sb^III^ concentration, namely, chromosomes 1 and 18 (from a disomic to a tetrasomic state) and chromosome 31 (from a tetrasomic to a hexasomic state). A singular case is chromosome 23, for which the reference estimate suggested a trisomic state, but which appears to exist in pentasomic state above 50 μM of Sb^III^ and then in tetrasomic state above 1600 μM. As previously reported for other *Leishmania* genomes, we obtained several intermediate somy estimates for certain chromosomes, which are likely to be associated with the phenomenon of mosaic aneuploidy.

In contrast to the extensive variation in chromosome somy observed in the laboratory-adapted strains, even before exposure to Sb^III^, we found little variation in the clinical isolates (Figure 2B). In these isolates, almost all chromosomes appear to be in a disomic state, with the notable exception of chromosome 31, which is in a tetrasomic state. Likewise, the phenomenon of intermediate somy estimates mentioned above was not detected in these isolates.

**Figure 3:**
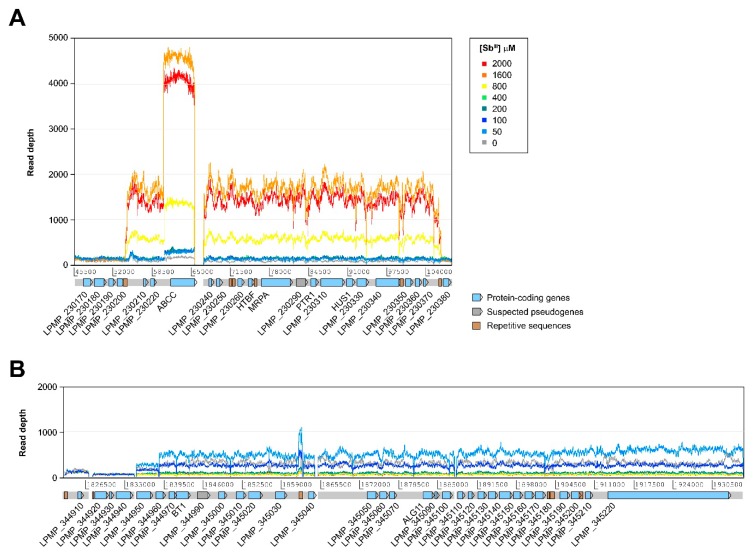
Relatively large chromosomal regions with differences in the estimated number of copies among different Sb^III^ concentrations. (**A**) Amplification of a region of ~53 kb of chromosome 23 overlapping the H-locus. (**B**) Region of ~101 kb from chromosome 34, suspected to be amplified in the form of a linear minichromosome. ABCC: ABC transporter, type C family; ALG11: α-1,2-mannosyltransferase; BT1: biopterin transporter 1; HTBF: H region terbinafine-associated resistance gene; HUS1: checkpoint protein HUS1; MRPA: multidrug resistance protein A; PTR1: pteridine reductase 1.

In addition to changes in the somy of certain chromosomes, we found evidence of amplification at several relatively large chromosomal regions in both the untreated reference strain and the treated samples (Figure S1, Table S3). Although many of these regions appear to be equally amplified before and after exposure to Sb^III^, at least two of them showed different copy number estimates at different Sb^III^ concentrations (Figure 3). One of these amplifications is located in chromosome 23, overlapping the region occupied by the H-locus (Figure 3A). This amplification appears to occur at concentrations above 800 µM of Sb^III^ and spans a region of ~53 kb, comprising 18 protein-coding genes. As expected, the amplified region contains gene LPMP_230280, coding for ABC transporter MRPA. Interestingly, the amplified region also appears to have a “nested” amplification of a locus encoding another ABC transporter of the type C family (LPMP_230230, ABCC), which is probably organized as a tandem gene array within the H-locus amplicons.

The second differentially amplified region observed in this study is located near one of the ends of chromosome 34, spanning approximately 101 kb and containing 28 protein-coding genes (Figure 3B). This amplification is likely to give rise to a linear minichromosome previously reported in *L. braziliensis* [22] and *L. panamensis* [23]. However, this minichromosome is only apparently present at Sb^III^ concentrations between 0 and 100 µM, with no evidence of amplification at higher concentrations. We found no evidence of the presence of this minichromosome or the H-locus amplification in the clinical isolates (results not shown).

**Figure 4:**
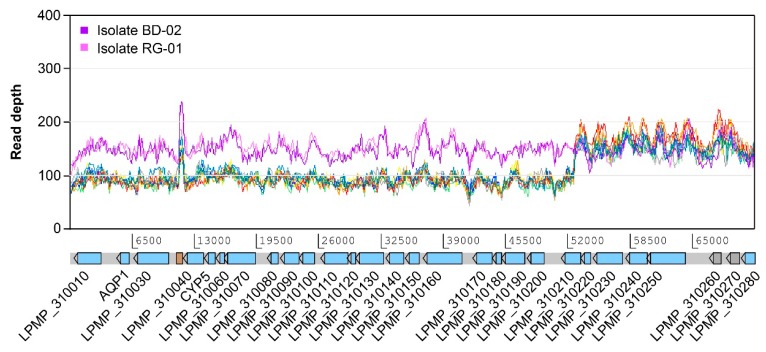
Relatively large subtelomeric deletion on chromosome 31. This deletion is estimated to be present in approximately half of the copies of this chromosome, spanning a region of 52 kb and affecting 20 protein-coding genes, including the one encoding aquaglyceroporin AQP1. The deletion is present in the PSC-1 reference strain even before exposure to Sb^III^, but it is not apparently present in clinical isolates BD-02 and RG-01. Except for those used for the isolates, colors in this figure follow the conventions used in Figure 3. CYP5: cyclophilin 5.

In addition to the subtelomeric amplification of chromosome 34, we observed several structural variations affecting the telomeric or subtelomeric regions of many chromosomes. Subtelomeric amplifications are present in chromosomes 8, 17, 19, 22, 26, 27, 31, and 34, although only those from chromosome 22 and 31 appear to have occurred after exposure to Sb^III^ and were subsequently maintained up to 2000 μM. Conversely, chromosome 31 also exhibited a uniform decrease in coverage in a region spanning 52 kb at one of its ends (Figure 4), suggesting the deletion of this region in some of the copies of the chromosome. This is the only example of a subtelomeric deletion we found in this study and it is uniformly present in all the treated samples and the untreated PSC-1 strain. Although this is not a total deletion, this variation potentially decreases the dosage of at least 20 genes to half of that for the rest of the genes in this chromosome. Remarkably, one of these is the AQP1 gene (LPMP_310020), which encodes for an aquaglyceroporin that is widely known to be involved in antimony resistance. As shown in Figure 4, this deletion is not present in the clinical isolates.

### 3.3. Variations at the Level of Individual Protein-Coding Genes

In order to identify copy number variations (CNVs) associated with individual protein-coding genes, we used a strategy based on the detection of local variations in read depth overlapping annotated gene models. As expected, we observed a uniform increase in read depth spanning several protein-coding genes in the Sb-treated samples and the untreated control, possibly associated with tandem gene arrays (Table S4). Excluding those genes located within the regions reported to be differentially amplified in the previous section (Figure 3A,B; Figure 4), the vast majority of the genes found to be locally amplified in the treated samples matched those previously reported for the strain PSC-1 [23], all with similar estimates of haploid copy number. A relatively small number of genes showed different copy number estimates at different Sb^III^ concentrations (Table 1), the majority of which were observed after exposure to the smallest concentration of 50 μM and were apparently maintained with similar estimates at higher concentrations. With very few exceptions, clinical isolates BD-02 and RG-01 also exhibited a similar set of amplified genes than the untreated PSC-1 strain (Table S5).

Many of the genes whose estimated copy number appears to have increased after exposure to Sb^III^ also seem to have a higher copy number in the clinical isolates, when compared to the untreated PSC-1 strain. These remarkably include genes widely known to be involved in parasite survival and virulence, such as LPMP_020120 (phosphoglycan-β-1,3-galactosyltransferase), LPMP_080610 (amastin-like protein), LPMP_080680 (tuzin), LPMP_090170/LPMP_190790 (autophagy-related protein 8), LPMP_100410/ LPMP_100440 (leishmanolysin), LPMP_130280 (α tubulin), LPMP_330860 (β tubulin), LPMP_170080 (elongation factor 1-α), LPMP_330370 (heat shock protein 83), and LPMP_331700 (peptidase M20/M25/M40). However, the number of copies estimated for some of these genes in the clinical isolates seems to be notably higher than in the treated samples. For instance, the gene encoding tuzin has an average estimated copy number of 27 in the treated samples and one of 37 in the isolates. Similarly, the gene coding for elongation factor 1-α appears to have 33 copies on average in the isolates versus 21 in the treated samples. In contrast, gene LPMP_311770, encoding a ubiquitin-fusion protein, appears to have fewer copies in the treated samples (27 on average) than in the untreated PSC-1 strain (32), but a notably higher number of copies in the clinical isolates (50 on average).

We also used a strategy combining two programs, GATK and SvABA, to detect relatively small variants such as single nucleotide variants (SNVs) and indels within protein-coding genes. Since the identification of such variants is technically difficult in regions subjected to different forms of genetic amplifications, we attempted to introduce the estimated somy for each chromosome as a parameter for the variant caller. However, SNVs predicted within genes in putative tandem arrays, whose reads tend to collapse in alignments, were flagged as heterozygotic and were not further considered. We found four genes harboring at least one SNV or indel that appear to have occurred after exposure to Sb^III^, namely, genes LPMP_120520, LPMP_120820, LPMP_323330, and LPMP_340960. All these genes encode hypothetical proteins of unknown function, except for LPMP_120820, which putatively codes for an *N*-ethylmaleimide reductase. The two genes from chromosome 12 have a SNV detected above 50 μM and maintained through all the concentrations subsequently evaluated. However, none of these SNVs were predicted to have a high impact in the function of the corresponding genes, since they are both associated with a putative missense mutation. Allele frequencies estimated with GATK for the SNVs detected in genes LPMP_120520, LPMP_120820, and LPMP_323330 provide additional evidence for the changes in somy reported for the corresponding chromosomes (Table S6), i.e., frequency for the two SNVs from chromosome 12 was 0.333, thus suggesting a trisomic state, while allele frequency for the SNV detected in LPMP_323330 changed from 0.5, the expected frequency for a disomic state, to 0.333 at 1600 μM.

On the other hand, distribution of small variants in genes LPMP_323330 and LPMP_340960 suggests that these genes may have internal amino acid repeats whose number of copies have varied during antimony treatment. Gene LPMP_323330 contains tetratricopeptide (TPR) repeat domains, IQ short calmodulin domains, and two domains of unknown functions, the latter present in at least two additional copies in concentrations above 800 μM of Sb^III^. Gene LPMP_340960 contains a Zinc finger domain of the CCCH type (Pfam: PF00642) and a repeat of nine units of the trinucleotide CAG, which is shortened to eight units due to the deletion of one repeated unit in the sample exposed to 2000 μM of Sb^III^.

## 4. Discussion

*Leishmania* is a kinetoplastid parasite with an extraordinary genomic plasticity. It is now well accepted that genomic changes occur frequently in this genus, commonly altering the number of copies of chromosomal regions and genes but maintaining global gene synteny. Since regulation of gene expression in *Leishmania* and the kinetoplastids does not appear to occur primarily at the level of transcription, many of these genomic changes may represent an adaptive advantage for the parasite, particularly if they alter the number of copies of critical genes to cope with changing environmental conditions. These forms of genetic amplification can be achieved in several ways, including the intrachromosomal increase in the number of copies of individual genes to give rise to tandem gene arrays, the extrachromosomal amplification of relatively larger regions to generate episomal amplicons, and the increase in somy of entire chromosome leading to aneuploid states that are well tolerated by the parasite. Here, we used whole genome sequencing (WGS) data to characterize and compare the genomic variations present in a laboratory-adapted strain of *L. panamensis*, seven samples derived from this strain exposed to increasing concentrations of Sb^III^, as well as two clinical isolates obtained from patients from two different Panama provinces, both with no sign of treatment failure. The main difference between strain PSC-1 and the two clinical isolates is that the later were recently isolated from human patients and have undergone two laboratory passages, while strain PSC-1 was isolated in 1994 and have undergone several passages.

Genomic changes have long been known to be critical for acquisition of resistance against pentavalent antimonials in *Leishmania*. Antimony resistance is a multifactorial process that involves several redundant mechanisms, all of them roughly aimed at inactivating the drug, decreasing its influx or increasing its efflux [8,31]. In fact, most of the studies addressing this topic in different *Leishmania* species have reported changes in the somy of individual chromosomes in strains with the Sb-resistant phenotype [9,10,13,14,15]. Here, we not only observed variations in chromosome somy in the final resistant parasites, but also noticed that these changes occurred actively during acquisition of resistance. Changes in somy started to occur after exposure to the lowest Sb^III^ concentration and some of these changes were maintained consistently through all the range of concentrations studied. Chromosomes 1, 18, and 31 showed a progressive increase in somy at increasingly higher Sb^III^ concentrations. Chromosome 23 also showed an increase in somy up to 800 µM of Sb^III^, but the somy decreased at higher concentration, a finding that may be associated with the occurrence of H-locus amplifications in samples above 800 µM of Sb^III^.

H-locus amplification is a type of episomal amplification that has been previously implicated in the acquisition of the antimony resistant phenotype. Here we observed the amplification of a region of ~53 kb overlapping the location of the original H-locus [32]. Amplification of the original region was shown to give rise to the so-called H circles, which contain two 30 kb inverted repeats derived from the amplified locus. The presence of these H circles was initially reported in *Leishmania* strains resistant to methotrexate [33,34] and was later implicated in resistance to antimonials [16,18]. Downing et al. [35] also reported the amplification of this locus in Sb-resistant *L. donovani* isolates; however, the region found to be amplified in this study was shorter (13.5 kb) and contained only four genes. Despite the evident variation in size, all forms of H-locus amplifications contain the MRPA gene encoding an ABC transporter mediating the incorporation of Sb-thiol complexes into intracellular vesicles [36]. These vesicles are thought to be involved in a secretory pathway that reduces the intracellular accumulation of Sb^III^, which can be considered a form of inactivation by sequestration [37].

A similar variation in size has been observed in a different type of episomal amplification well studied in *Leishmania*, this one associated with a different locus in chromosome 34 and called LD1. In contrast to H-locus amplifications, LD1 amplicons have not been associated with antimony resistance, but with an increased fitness for the parasite due to the presence of a gene encoding a biopterin transporter (BT1). Here, we found evidence for the presence of an LD1 amplicon resembling a linear minichromosome, previously described for *L. braziliensis* strain M2903 [22]. This amplicon was found in our reference strain PSC-1 and it was apparently maintained in this strain after treatment with Sb^III^ up to a concentration of 100 µM. The loss of this amplicon at higher Sb^III^ concentrations may be associated with the later occurrence of other amplifications putatively involved in the Sb-resistant phenotype. Among these, the H-locus amplification and two subtelomeric amplifications found above 100 µM of Sb^III^, the later respectively located in chromosome 22 (~97 kb, 26 genes) and chromosome 31 (~150 kb, 45 genes). However, further studies are required to find if these amplifications are in fact associated with antimony resistance or are just stochastic changes, a product of the highly plastic nature of the *Leishmania* genome. In fact, Patino et al. [15] recently reported the presence of a ~50 kb amplification in the subtelomeric region of chromosome 27 of Sb-resistant *L. panamensis* mutants, which we did not observe in our treated samples.

We also noticed the deletion of a ~52 kb subtelomeric region of chromosome 31 in both the treated samples and the untreated strain PSC-1, but not in the clinical isolates. This deletion can be considered partial, since it appears to affect roughly half of the copies of this chromosome, which was estimated to be at least tetrasomic in all the samples included in this study. A similar terminal deletion of chromosome 31 has been previously reported for Sb-resistant mutants of *L. major* [13] and *L. guyanensis* [38]. In all cases, the deleted region contains the AQP1 gene encoding a specific aquaglyceroporin that imports Sb^III^ into amastigotes [19]. Since the untreated PSC-1 strain has a notably higher EC_50_ value than the susceptible clinical isolates, it is likely that even a decrease in the number of copies of this gene, rather than its total loss or inactivation, may give the parasite an adaptive advantage in the presence of Sb^III^.

In addition to changes associated with entire chromosomes or relatively large chromosomal regions, it is now well known that changes at the level of certain genes are also associated with the Sb-resistant phenotype. For instance, Monte-Neto et al. [14] reported a higher gene dosage for the MRPA gene in four Sb-resistant *L. guyanensis* mutants, but due to tandem amplification of this gene, rather than the extrachromosomal amplification of the H-locus. This form of intrachromosomal amplification, leading to tandem gene arrays, have been reported in all *Leishmania* genomes sequenced to date, although the set of genes that are amplified can be slightly different. Here, we observed that the vast majority of the genes that were found to be locally amplified in reference strain PSC-1 maintained their organization in suspected tandem gene arrays after exposure to Sb^III^, suggesting that these are long-term or stable amplifications, conserved during evolution regardless of environmental stress. However, we indeed observed differences in the estimated haploid copy numbers for many of these genes, the majority of them occurring after exposure to the lowest Sb^III^ concentration of 50 µM. A remarkable case is that of gene LPMP_230230, encoding a type C ABC transporter (ABCC), which appears to form a tandem gene array within the H-locus amplicons, resembling a form of “nested” amplification.

Surprisingly, we found notable less genomic variations in the clinical isolates, when compared to the treated samples and the untreated PSC-1 strain. In these isolates, there is almost no occurrence of subtelomeric or episomal amplifications and changes in the somy of entire chromosomes (with the exception of chromosome 31). A reduction in aneuploidy in samples isolated from animal hosts has been previously reported for other *L. donovani* species [11,12,39]. The predominant form of genetic amplification in our clinical isolates appears to be tandem gene arrays, with some genes exhibiting remarkably higher copy number estimates than the laboratory-adapted samples.

Our findings suggest that the set of genomic amplifications occurring in a *Leishmania* population may be balanced by the compromise between availability of precursors required for DNA synthesis and the advantage these amplifications may represent for the parasite in terms of fitness, under certain environmental conditions. For instance, at high Sb^III^ concentrations in vitro, the somy of the already tetrasomic chromosome 23 was observed to decrease, while the number of copies of the putatively episomic H-locus amplification increased. Occurrence of these H-locus amplifications also appears to be associated with the loss of the minichromosome deriving from chromosome 34. In contrast, this minichromosome, which is thought to give an adaptive advantage related to folate metabolism, appears to be present in vitro in the reference strain but only in absence or low concentrations of Sb^III^. While none of these amplifications were found in the clinical isolates, notably higher numbers of copies were observed for certain genes widely known to be involved in infection and virulence. These findings contribute to emphasize the relevance of rapid changes at the DNA level in a pathogenic parasite with almost no transcriptional regulation of gene expression.

Although the balance between precursor availability and response to environmental stress could be a potential explanation of changes observed in this study, we do not rule out other possible scenarios. For instance, it has been shown in other unicellular eukaryotes that large changes in gene dosages may produce an array of potential problems in cells, including a response similar to those triggered by environmental stress [40,41] and slower growth and proliferation [42,43,44]. Additionally, aneuploidy in eukaryotes may cause metabolic and proteotoxic stress [45]. Also, variations in gene copy number have been shown to cause a dramatic effect in fitness, as is the case of the β-tubulin gene in *Saccharomyces cerevisiae* [46]. Therefore, since many of the genes in amplified regions are of unknown function, it may be possible that amplification at certain genomic regions may also increase unintended gene activities, ending up in a reduction of parasite fitness. Then, in an attempt to limit these adverse effects, secondary or compensatory genomic variations may also occur. More studies will be necessary to establish if some of the variations observed here in *L. panamensis* may indeed have a compensatory effect.

## Figures and Tables

**Table 1:** Protein-coding genes with different haploid copy number estimates among the samples included in this study ^1^.

Gene	Suspected Function	Estimated Haploid Copy Number									
		PSC-1	Treated Samples [Sb^III^] µM							RG-01	BD-02
			50	100	200	400	800	1600	2000		
LPMP_020120	Phosphoglycan β-1,3-galactosyltransferase	2	3	3	3	3	3	3	3	4	5
LPMP_040170	Hypothetical protein	10	14	14	14	14	14	13	13	18	14
LPMP_040280	β-fructofuranosidase	3	4	4	4	4	4	4	4	2	5
LPMP_050490	ATPase α subunit	3	4	4	4	4	4	3	3	4	6
LPMP_060560	60S ribosomal protein L23a	3	2	2	2	2	2	2	2	2	2
LPMP_080610	Amastin-like protein	14	19	17	17	17	18	16	16	14	21
LPMP_080680	Tuzin	22	30	27	27	26	28	27	26	39	34
LPMP_080890	Hypothetical protein	2	1	1	1	1	1	1	1	1	1
LPMP_090170	Autophagy-related protein 8 (ATG8)	13	19	20	19	20	21	19	19	20	16
LPMP_100410	GP63, leishmanolysin	9	12	12	11	11	11	10	11	13	10
LPMP_100440	GP63, leishmanolysin	6	8	8	8	8	8	7	7	8	7
LPMP_130280	α-tubulin	19	21	22	22	23	22	22	22	26	24
LPMP_160880	Flagellar calcium-binding protein	3	2	2	2	2	2	2	2	2	2
LPMP_170080	Elongation factor 1-α	15	21	21	21	21	22	20	20	33	34
LPMP_190790	Autophagy-related protein 8 (ATG8)	10	15	15	16	14	16	14	14	11	16
LPMP_311770	Ubiquitin-fusion protein	32	28	27	27	27	28	22	23	51	50
LPMP_330370	Heat shock protein 83	12	14	14	14	14	14	13	13	16	12
LPMP_330860	β-tubulin	34	45	43	43	43	44	41	41	41	34
LPMP_331700	Peptidase M20/M25/M40	12	15	14	15	15	14	14	14	15	12

^1^ This table does not include the genes located within relatively large regions suspected to be episomic amplifications, as well as other forms of subtelomeric variations, all of which also have a different gene dosage.

## Supplementary Materials

The following are available online at https://www.mdpi.com/2073-4425/10/11/838/s1, Figure S1: Distribution of sequenced reads from evaluated samples aligned against the 35 *L. panamensis* reference chromosomes, Table S1: Estimates of chromosome somy for all the samples included in this study, Table S2: Statistical significance of treated samples predicted to have changes in somy, Table S3: Genomic variations involving relatively large chromosomal region in the antimony-treated *L. panamensis* samples, Table S4: Genes estimated to be present in two or more copies in the antimony treated samples and the untreated PSC-1 strain, Table S5: Genes estimated to be present in two or more copies in the clinical isolates. Table S6: Single nucleotide variants (SNVs) that appear to have occurred after exposure to Sb^III^.
