# Supplementary material for: Environmental Conditions May Shape the Patterns of Genomic Variations in Leishmania panamensis
Source: Genes (Basel). 2019 Oct 24;10(11):838. doi: 10.3390/genes10110838 (PMC6896075; doi:10.3390/genes10110838)
Supplement: Supplementary file 1 [file genes-10-00838-s001.zip › Figure S1.pdf]

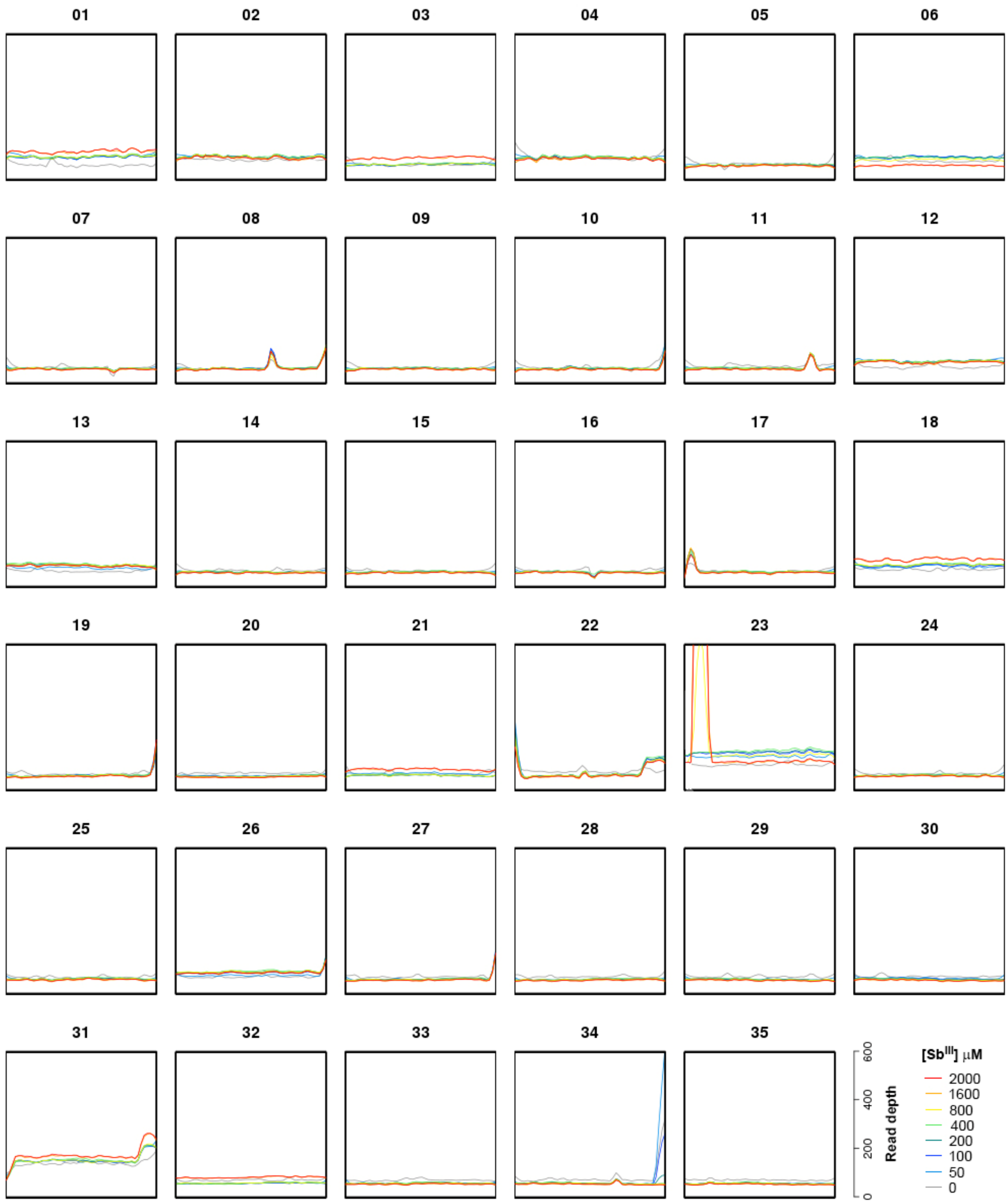

Figure S1. Distribution of sequenced reads from evaluated samples aligned against the 35 *L. panamensis* reference chromosomes. Plots show the read depth per position averaged over a 50 mer window and smoothed with the loess function of the R package. For a better visual comparison, *x* axis scale does not correlate with the actual length of the chromosome.
